# An Atypical Etiology of Acute Pericarditis: A Case Report

**DOI:** 10.7759/cureus.13440

**Published:** 2021-02-19

**Authors:** Shelley Persaud, Bir Singh, Debra Angelo

**Affiliations:** 1 Internal Medicine, HCA Medical Center of Trinity, Trinity, USA

**Keywords:** etiology of pericarditis, acute pericarditis, pericarditis, atypical pericarditis, tejocote root, tejocote, dietary supplement, diet, side effects, ekg changes

## Abstract

Acute pericarditis is caused by inflammation of the pericardial sac and is characterized by sharp and pleuritic chest pain, which is accompanied by a pericardial friction rub and serial electrocardiogram (ECG) changes. Although there are many known etiologies of pericarditis, we present the first known case of a herbal supplement, the Tejocote root, causing acute pericarditis in a previously healthy 23-year-old female. Her ECG showed t-wave inversions that resolved with colchicine and non-steroidal anti-inflammatory drug therapy. Prior studies have demonstrated that it can cause adverse cardiovascular effects, but this is the first documented case of the Tejocote root causing acute pericarditis. This case report reinforces the importance of inquiring about supplements patients may be taking, especially when looking for the etiology of pericarditis.

## Introduction

Acute pericarditis is caused by the inflammation of the pericardium, a fibroelastic sac encompassing the heart. The various etiologies of acute pericarditis include viral infections, autoimmune diseases like systemic lupus erythematosus (SLE), malignancy, postoperative issues, especially those that emerge after coronary artery bypass grafting (CABG), uremia from renal failure, trauma, chemotherapy drugs, or radiation therapy [1]. Patients typically present with a complaint of sharp, central chest pain, which is worsened by lying supine and alleviated by leaning forward. A pathognomonic finding is a pericardial friction rub, which has three components corresponding to atrial systole, ventricular systole, and ventricular diastole, but this may not always be present. The pericardial friction rub is present anywhere from 35 to 85% of the time and depends on the frequency of auscultation as it is transient [2]. Electrocardiogram (ECG) is very useful in the diagnosis of acute pericarditis with abnormalities found in approximately 90% of cases, and, depending on the stage, diffuse concave-upward ST-segment elevation with PR depression may be seen [3]. The lack of reciprocal ST-segment changes differentiates acute pericarditis from acute myocardial infarction.

The Tejocote root is a herbal root derived from the Crataegus tree, which is native to the mountains of Mexico and some parts of Guatemala. The fruit has long been used in traditional Mexican culture for the treatment of cough, urinary retention, and breathing difficulty [4]. Currently, the Tejocote root is commonly sold as a weight-loss supplement under the brand name Alipotec at major retailers in the United States. A presumed mechanism behind its ability to cause weight loss is the high pectin content within the root, which has been associated with early satiety [5]. Adverse effects include myalgias and gastrointestinal upset. Previous studies have also demonstrated that it can cause second-degree heart block and immune thrombocytopenia [6]. In this report, we present a case of the Tejocote root causing acute pericarditis.

## Case presentation

**Figure 1:**
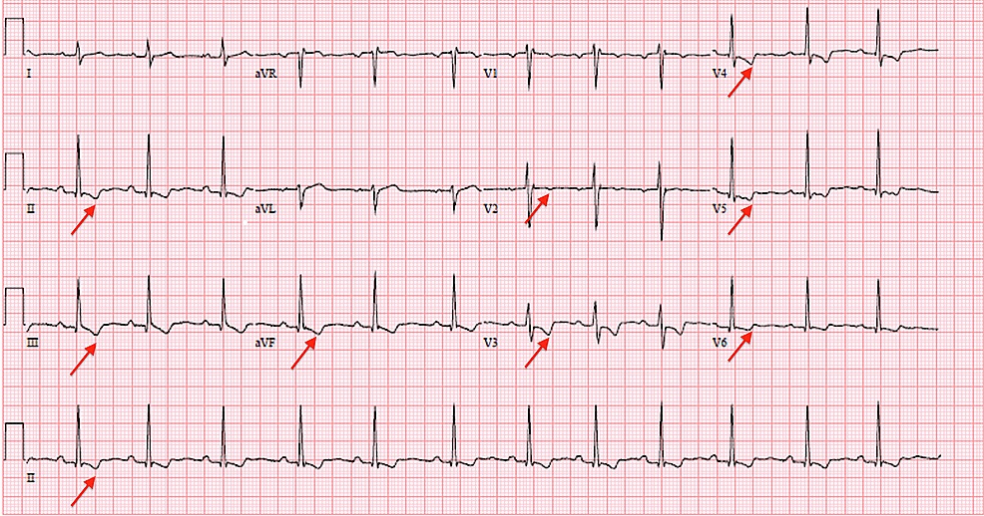
Initial ECG showing t-wave inversions in the inferior and anterolateral leads (red arrows) ECG: electrocardiogram

**Figure 2:**
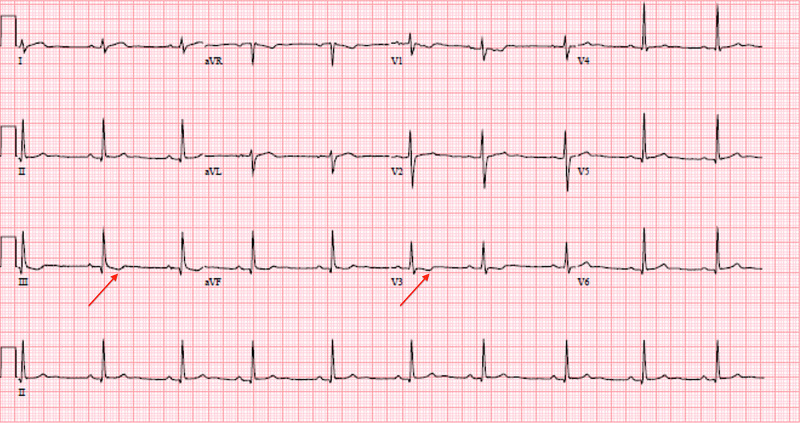
Repeat ECG on hospital day three showing t-wave inversions in leads III and V3 only (red arrows) ECG: electrocardiogram

**Figure 3:**
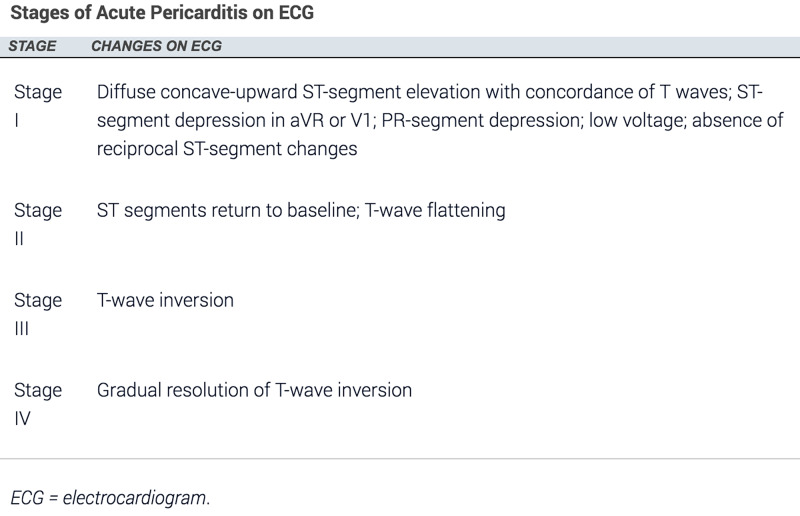
ECG changes in acute pericarditis

A 23-year-old female presented to our emergency department with chest pain for the past nine hours. The pain was substernal, constant, pressure-like, 6/10 in severity, and radiating to her back. It was aggravated by lying flat on her back and alleviated by sitting up. She admitted to taking a Tejocote root supplement for weight loss four days prior to the onset of chest pain, which had caused her to have loose stools, increased thirst, and fatigue. She denied any sick contacts. The patient had no significant past medical history, such as pre-existing cardiovascular disease, autoimmune conditions [such as rheumatoid arthritis (RA) or SLE], renal failure, malignancy, or a preceding viral illness. The only prescribed medication she was on was an oral contraceptive pill, and she denied taking any other oral supplements. Initial and subsequent ECGs are presented in Figure 1 and Figure 2, and the stages of acute pericarditis on ECG are shown in Figure 3 [3]. 

Initial vitals were as follows: temperature of 98.6 °F, blood pressure of 198/107 mmHg, pulse rate of 80 beats/minute, respiratory rate of 16 breaths/minute, and oxygen saturation of 100% on room air. Physical examination showed no pericardial friction rub. ECG showed t-wave inversions in the inferior and anterolateral leads (Figure 1).

Transthoracic echocardiogram showed an ejection fraction of 55%, no wall motion abnormalities, no pericardial effusion or thickening, and normal diastolic function.

Acute coronary syndrome was ruled out due to negative troponin levels and ECG findings. Based on her symptoms and ECG findings, we diagnosed her with acute pericarditis and started her on intravenous Toradol 15 milligrams every eight hours and colchicine 0.6 milligrams daily. Cardiology was consulted and they agreed with the diagnosis and management. Her chest pain resolved completely within two days. On hospital day three, repeat ECG showed t-wave inversions in leads III and V3 only (Figure 2).

Based on the guidelines adopted by the American College of Cardiology for treating the first occurrence of acute pericarditis, our patient was discharged with a three-month course of colchicine [7]. She was instructed to follow up in the cardiology clinic; however, the patient did not follow up since her chest pain completely resolved. Figure 3 shows the progression of acute pericarditis on ECG [3].

## Discussion

In addition to acute pericarditis, the Tejocote root has been shown to cause other potentially reversible adverse effects [5,6]. In one case report, a healthy 16-year-old female who had taken her mother’s weight-loss supplement, the Tejocote root, suffered from drowsiness, vomiting, and diarrhea. She developed Mobitz type I second-degree heart block. Given the root’s structural similarity to digitalis, it cross-reacted with commercial digoxin assays, causing falsely elevated digoxin levels [5]. Surprisingly, 29 hours after the ingestion, the patient reentered normal sinus rhythm. It is unclear how the Tejocote root caused reversible second-degree heart block, and further studies are needed to explore the underlying mechanism.

In another case report, a 51-year-old female with no history of bleeding disorders took the Tejocote root for weight loss over six weeks, which caused her platelets to drop as low as 25,000/microliter [6]. Infectious etiologies such as hepatitis B and C, HIV, Epstein-Barr virus, and Helicobacter pylori were ruled out. Workup for an inflammatory and/or autoimmune illness was negative. When treated with prednisone, her platelet levels increased initially but then dropped, and did not return to normal levels until she stopped taking the Tejocote root. It is unclear how the Tejocote root causes reversible thrombocytopenia and why there was a lack of sustained improvement with oral prednisone. Further research is needed to probe into the underlying mechanism.

Interestingly, stopping the use of the Tejocote root in both cases reversed the adverse side effects. This could potentially be related to the half-life and clearance of the root. Evidently, further studies into the substance's pharmacokinetics are required.

Based on our literature review, this is the first documented case of the Tejocote root causing acute pericarditis. There are multiple atypical features associated with this case presentation. Though more than half of the patients develop the typical ECG features listed in Figure 3, they occur over a period of weeks [3]. Though our patient presented with chest pain lasting for nine hours, her initial ECG was consistent with stage III of acute pericarditis. Furthermore, her ECG changes nearly resolved with one day of therapy with Toradol and colchicine. The patient did not follow up in the cardiology clinic since her chest pain completely resolved; it would have been interesting to see the complete resolution of the ECG changes.

## Conclusions

It is important for clinicians to inquire about the drugs or supplements a patient may be taking as they can have unexpected adverse effects. This is especially applicable when evaluating a young patient with pericarditis as herbal weight-loss supplements containing the Tejocote root can result in acute pericarditis. Also, in our case and in both of the case studies we found in the literature, adverse effects of the Tejocote root resolved once the patients discontinued its use; it would be interesting to explore if consuming it for a longer duration could lead to irreversible outcomes.
